# Development and Validation of four Nitrosamine Impurities Determination Method in Medicines of Valsartan, Losartan, and Irbesartan with HPLC-MS/MS (APCI)

**DOI:** 10.22037/ijpr.2021.115102.15195

**Published:** 2021

**Authors:** Mikhail Khorolskiy, Galina Ramenskaya, Alexander Vlasov, Oleg Perederyaev, Nataliya Maslennikova

**Affiliations:** a *I.M. Sechenov First Moscow State Medical University of the Ministry of Health of the Russian Federation (Sechenov University), Russia. *; b *Federal State Budgetary Institution Scientific Centre for Expert Evaluation of Medicinal Products, Russia.*

**Keywords:** Nitrosamine, Valsartan, Losartan, Irbesartan, HPLC-MS/MS

## Abstract

Since 2018, regulation and control of genotoxic nitrosamine impurities levels have been a mandatory quality and safety characteristic for various drugs. The main issue of nitrosamine determination in drugs is a low sensitivity of the existing methods and a continuously extending list of controlled compounds. The reason is the low safe daily dose of these impurities and chromophores’ absence within their structure. Development and validation of a method for nitrosamine impurities (regulated by the regulatory authorities) determination in Valsartan, Losartan, and Irbesartan using high-performance liquid chromatography with mass spectrometry detection. An Agilent Infinity II chromatographic system with a mass spectrometric detector (MSD 6460 Triple Quad) and atmospheric pressure chemical ionization was used in this study. During the development of a method, the optimal conditions for chromatographic separation (composition of mobile phases, gradient parameters) were selected, as well as the parameters of mass spectrometric detection were optimized. The usage of chemical ionization made it possible to achieve the method’s maximum sensitivity concerning the studied nitrosamines, and the optimized parameters of mass spectrometric detection made it possible to get rid of the matrix effect. The absence of additional stages of purification and concentration can significantly reduce the total time of the analysis, which is a significant advantage of nitrosamine’s advanced determination method. The resulting method was validated for specificity, linearity, LOQ, LOD, accuracy, and precision. Resulting method met all acceptance criteria and can be used for routine quality control of Valsartan, Losartan, and Irbesartan pharmaceutical substances.

## Introduction

Since 2018, the leading regulatory authorities (EMEA, FDA) have been requiring that, for some angiotensin II receptor blocker (belongs to a class of medicines called sartans) and, later, some other medicines (histamine antagonists, metformin, nitroglycerin), regulation and control of nitrosamine impurities levels are a mandatory quality and safety characteristic (1-3). 

Nitrosamine impurities are compounds containing a nitroso group at the dialkyl-substituted amine group. In Valsartan, Losartan, and Irbesartan substances, these impurities result from the reaction of solvent impurities (used for synthesis) and nitrite ions in the acidic environment (4). As these compounds are used to synthesize various organic substances, all drugs should be checked for nitrosamines if their structure is at high risk of nitrosamine formation or produced using similar synthesis steps. This conclusion is supported by screening studies that demonstrated the presence of nitrosamines in *e. g.* Ranitidine and Metformin (5). 

The primary danger from nitrosamine impurities is that they belong to compounds possessing “potential genotoxicity” meaning the availability of reliable information on genotoxicity obtained during animal studies and lack or insufficient information on toxicity for humans (6). 

Nitrosamine impurities are included in the “cohort of concern” compounds; that means that the general approach to the acceptable concentration determination using the toxicological value threshold is not applicable in this case (7). The acceptable interim concentrations of major nitrosamine impurities for active pharmaceutical ingredients are presented on the FDA website (8). These values are based on animal toxicity studies (9,10). The safety levels are as follows: NDMA – 96 ng/day, NDEA – 26.5 ng/day, NMBA – 96 ng/day, NEIPA – 26.5 ng/day. Iupac names of the target nitrosamines:

NDMA - N, N-Dimethylnitrous amide

NDEA - N, N-Diethylnitrous amide

NMBA - 4-[methyl(nitroso)amino]butanoic acid

NEIPA - N-ethylpropan-2-amine

The concentrations, which should be quantified in drugs taking into account daily doses of active substances, are low – 0.08 to 3 ppm and are difficult to quantify with usual drug quality control methods. Moreover, the structural formula of nitrosamines (demonstrating the highest risk of presence in a drug) does not contain any distinct groups allowing detecting them with the required sensitivity when using traditional spectral analysis methods. The only exception is N-nitro-4-methyl-aminobutyric acid (NMBA), which demonstrates high signal intensity when using various methods because of the carboxyl group in its composition. 

Thus, the drug analysis methods used for routine quality control do not have sufficient sensitivity and selectivity to determine nitrosamine impurities in drugs. The majority of existing nitrosamine impurities determination methods use chromatographic separation of components under discussion (mostly HPLC) with MS detection (11-13). The single quadrupole detection methods can determine the desirable nitrosamine concentrations only at the detection limit. Over the previous three years, multiple methods have been developed both for individual impurities and for drugs. Nevertheless, with consideration for equipment availability, product lines, and regulatory requirements, the issue of universal control methods is still open. Only four nitrosamine impurities (NDMA, NDEA, NMBA, NEIPA) have been included in regulatory requirements for their screening and monitoring in sartan medicines; however, over 12 compounds from this group have been studied (14, 15). 

Regulatory authorities, FDA, EMA developed methods for nitrosamine determination in Valsartan, Losartan, and Irbesartan substances (16, 17). FDA method based on the rare and expensive equipment such as LC-HRMS. Moreover, this method is intended to simultaneously determine six nitrosamine impurities, while only four nitrosamines are regulated. Since a complex multi-component matrix is absent, using sensitivity level equipment such as a mass-spectrometer with high resolution is unnecessary. Method EMA is used for simultaneous determination of two nitrosamine impurities (NDMA, NDEA), while the regulatory authorities also regulate NMBA and NEIPA content. Few complementary methods can only check the quality of the substances by nitrosamine impurities presence. It makes routine quality control longer and time-consuming.

The main aim of the presented study was to develop and validate a routine method for determining four regulated genotoxic nitrosamine impurities in pharmaceutical substances Valsartan, Losartan, and Irbesartan with HPLC-MS/MS.

## Experimental

*Reagents, Standards, and Samples*

The following reagents were used in the study:

Acetonitrile (for HPLC, gradient grade, ≥99.9%, Sigma-Aldrich, Cat. No. 439134)

Methanol (for HPLC, gradient grade, ≥99.9%, Sigma-Aldrich, Cat. No. 34885)

Formic acid (ACS reagent, ≥96%, Sigma-Aldrich, Cat. No. 695076) 

Purified water, Milli-Q^®^ Advantage A 10

Ammonium formate (eluent additive for LC-MS, LiChropur™, Supelco, Cat. No. 70221)

Nitrosamine impurity standards:

N-Nitrosodimethylamine (NDMA), certified reference material, Supelco, Cat. No. PHR2407;

N-Nitrosodiethylamine (NDEA) 98%, Santa Cruz Biotechnology, Inc, Cat. No. sc-212252;

N-(methyl)(nitroso)amino)butanoic acid (NMBA) min 98% (TLC), BIOSYNTH & CARBOSYNTH, Cat. No. FN26379;

N-Nitroso-isopropylethylamine (NEIPA) SimSonPharma Cat. No. OT40150000;

Pharmaceutical substances as standards were obtained from the Cymit Quimica catalog:

Valsartan Cat. No. 7W-GP3294

Losartan potassium Cat. No. 3B-L0232

Irbesartan Cat. No. 3B-I0859

Pharmaceutical substances for method approbation:

Valsartan (Zhejiang Huahai Pharmaceutical) Batch № 10250-181102

Losartan (Zhejiang Tianyu Pharmaceutical) Batch № 10100-180322-04

Irbesartan (Zhejiang Huahai Pharmaceutical) Batch № 10050-180205

*Preparations of solutions*

Preparation of stock solutions: Place 1 mg of a nitrosamine impurity standard into a 100 mL volumetric flask, accurately weighed, add to the volume with methanol, and thoroughly mix for 1 min until dissolved.

Preparation of standards (NDMA, NDEA, NMBA, NEIPA)

Add approximately 10 mL of solvent to a 100 mL volumetric flask. Add 2 mL of each impurity stock solution. Vortex for 1 min. Add to the volume with solvent and mix thoroughly: (NDMA concentration: 0.2 µg/mL; NDEA concentration: 0.2 µg/mL; NMBA concentration: 0.2 µg/mL; NEIPA concentration: 0.2 µg/mL). The preparation of calibration standards for impurities is described in Table 1.

*Model mixtures preparation*

Prepare 18 model mixtures for each active substance (Valsartan, Losartan, Irbesartan) using each calibration standard. Put a sample weight equivalent to the maximum daily sartan intake into a centrifuge tube (Valsartan 320 mg, Losartan 150 mg, Irbesartan 300 mg). Add 5 mL of calibration standard with the required concentration. Vortex thoroughly for 1 min and sonicate (100W; 35kHz) the mix for 5 min using an ultrasonic bath. Add 5 mL of the same calibration solution and mix for 2 min. Sonicate for 5 min. Centrifuge for 7 min at 2,050 g. Filter 2 mL of supernatant using a 0.45-micron filter. Prepare model mixtures with nitrosamine concentrations 0.2; 0.4; 0.6; 0.8; 0.9; 1.1 ng/mL. 

*Equipment and Analysis Parameters*

The NDMA, NDEA, NMBA, NEIPA impurities were identified and quantified using high-efficiency liquid chromatograph Agilent Infinity II with MS detector (MSD 6460 Triple Quad). Column – Agilent InfinityLab Poroshell 120 EC-C18, 50 mm, 3.0 mm, 2.7 µ (cat. 699975-302). Chromatographic separation parameters and settings of mass spectrometry detection are presented in Table 2.

## Results and Discussion

*Method Development*

The development of the method for identifying and quantifying nitrosamines (NDMA, NDEA, NMBA, NEIPA) was divided into several steps, with the significant steps being the development of chromatographic separation parameters settings of mass spectrometry detection.

An essential criterion of chromatographic separation was the possibility to separate the pharmaceutical substance from nitrosamines under discussion. This criterion significantly enhances the method’s selectivity and reduces the probability of impaired MS detector sensitivity due to contamination with high concentrations of non-target components (pharmaceutical substances). Another valuable parameter of chromatographic separation is its duration (less than 10 minutes). This fact allows using this method for routine quality control, thus for many samples within a limited time. 

The chromatographic column was selected with the help of publications describing the development of nitrosamine analysis methods in pharmaceutical substances (14). We selected a short column with sufficient efficiency for mid-polar compounds. Solvents used as a mobile phase for chromatography were selected experimentally by analyzing nitrosamine standards. The combination described in Experimental is the most efficient for separating nitrosamines under discussion and pharmaceutical substances. 

**Figure 1:**
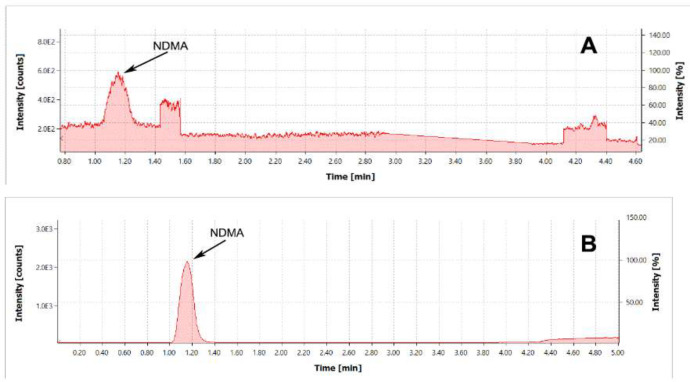
(A) Chromatogram of NDMA standard with the concentration of 0.2 µg/mL. TIC MRM (75.1 -> 58.1) Type of ionization – APCI. Mobile phase composition: formic acid 0.1 % in methanol. (B) Chromatogram of NDMA standard with the concentration of 0.2 µg/mL. TIC MRM (75.1 -> 58.1) Type of ionization – APCI. Mobile phase composition: formic acid 0.1% in methanol and ammonium formate 10 mmol/L water

The study demonstrated that the addition of substances that boost ionization improves method sensitivity and reproducibility. For boosting the ionization process, formic acid (with the concentration in the mobile phase of 0.1%) and ammonium formate (with a concentration of 10 mmol/L) were added to the methanol and water as follows. Formic acid (with a concentration in the mobile phase of 0.1%) was added to methanol in the mobile phase. Typical chromatograms of the NDMA standard are given in Figure 1. 

As shown in Figure 1, the addition of substances that boost ionization significantly improves the NDMA response. 

Following the selection of conditions and parameters for chromatographic separation, the next important step is selecting and optimizing MS detection parameters. Nitrosamine standard solutions were analyzed in the Scan mode to identify parent ions. Selected parent ions were used to choose optimal MRM transitions. Stock standard solution of nitrosamine impurities (NDMA, NDEA, NMBA, NEIPA) with a concentration of 0.2 µg/mL was analyzed in the Scan mode for optimal MRM transition selection. The highest and largest peak areas were recorded with the analysis settings described in Experimental*.* The obtained results almost wholly correlate with the results published in scientific studies describing the development and validation of nitrosamine determination methods in pharmaceutical substances (15). For different works of obtained MS detection parameters, the nitrosamine solution with a 1.1 ng/mL concentration was analyzed using MassHunter Workstation Software Optimizer, version B.07.01. Once the workout of MS detection parameters had been completed, chemical ionization parameters were optimized. The highest sensitivity was achieved with the corona current of 4 µA. This parameter correlates with the data from publications.

**Figure 2:**
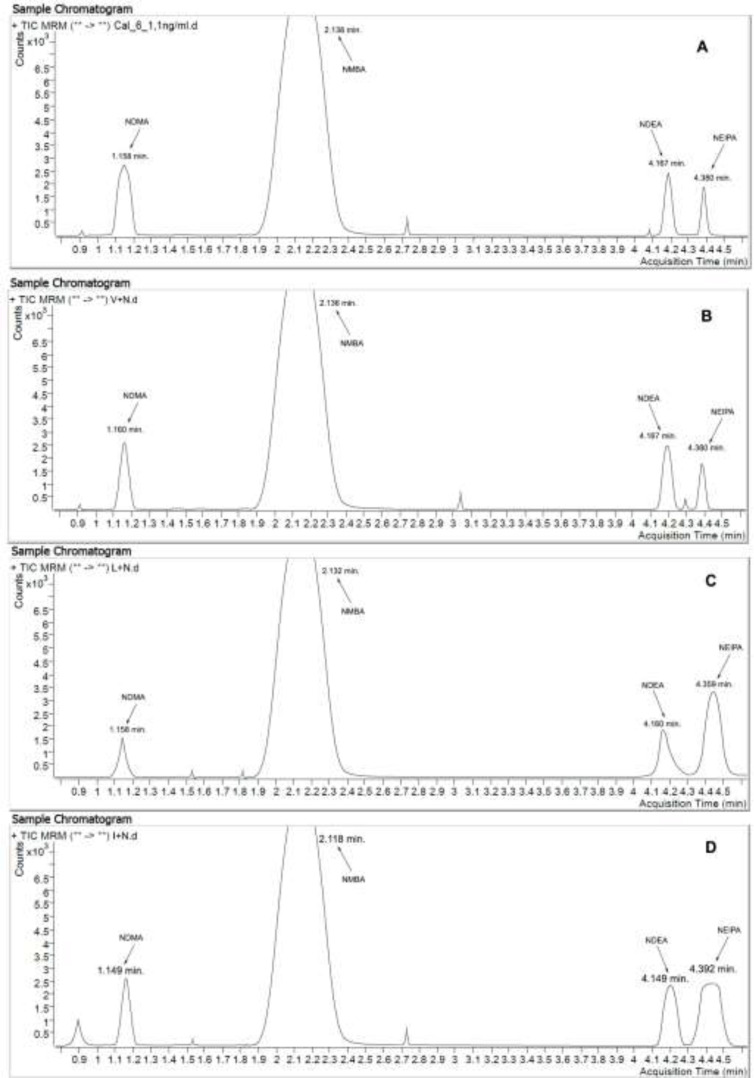
(A) Chromatogram of standard mixture with addition of nitrosamines (concentration: 1.1 ng/mL). TIC MRM (75.1 -> 58.1; 147.1 -> 117.1; 103.1 -> 29.1; 117.1 -> 75.1). (B) Chromatogram of Valsartan model mixture with addition of nitrosamines (concentration: 1.1 ng/mL). TIC MRM (75.1 -> 58.1; 147.1 -> 117.1; 103.1 -> 29.1; 117.1 -> 75.1). (C) Chromatogram of Losartan model mixture with addition of nitrosamines (concentration: 1.1 ng/mL). TIC MRM (75.1 -> 58.1; 147.1 -> 117.1; 103.1 -> 29.1; 117.1 -> 75.1). (D) Chromatogram of Irbesartan model mixture with addition of nitrosamines (concentration: 1.1ng/mL). TIC MRM (75.1 -> 58.1; 147.1 -> 117.1; 103.1 -> 29.1; 117.1 -> 75.1)

A chromatogram of Valsartan, Losartan, and Irbesartan model mixture with the concentration of 1.1 ng/mL of nitrosamines under discussion and in the settings described in Experimental presented at Figure 2.

As the method meets the acceptance criteria, it was validated. The method was validated as per FDA guidelines for analytical method validation. The method was validated for all major validation parameters (LOQ, LOD, linearity, precision, accuracy).

*Method Validation*

*Specificity*

Method specificity was confirmed with the analysis of a mixture of Valsartan, Losartan, and Irbesartan standards. The primary acceptance criterion for specificity was the lack of foreign peaks and pharmaceutical substance peaks at the point of nitrosamine detection (Figure 4).

**Figure 3:**
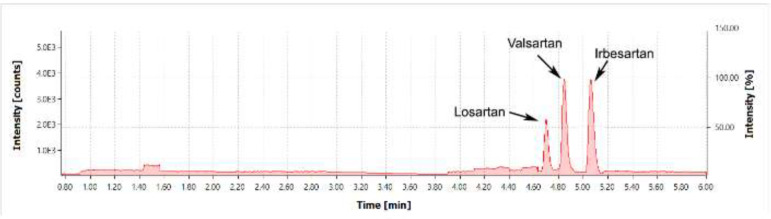
Chromatogram of a mixture of Valsartan, Losartan, and Irbesartan standards obtained in the SCAN mode, range: 150-500 m/z

Figure 3 shows a chromatogram of a mixture of Valsartan, Losartan, and Irbesartan standards. Because of the retention time of the Irbesartan peak, the chromatographic gradient was adjusted as follows: 

0 – 1.5 min, as per the gradient in Experimental

1.5 – 6 min. Phase ratio – 5:95.

*Limit of Detection (LOD)*

To evaluate the limit of detection for the established method, Valsartan, Losartan, and Irbesartan model mixtures were analyzed with nitrosamine concentrations of 0.2; 0.4; 0.6; 0.8; and 1.1 ng/mL, respectively. The primary acceptance criterion for the limit of detection was a signal-to-noise (S/N) ratio of 3. Experiments helped in setting the method limit of detection of 0.2 ng/mL. With this concentration, the mean S/N ratio obtained with three consecutive measurements was 3.7.

*Limit of quantification (LOQ)*

**Figure 4:**
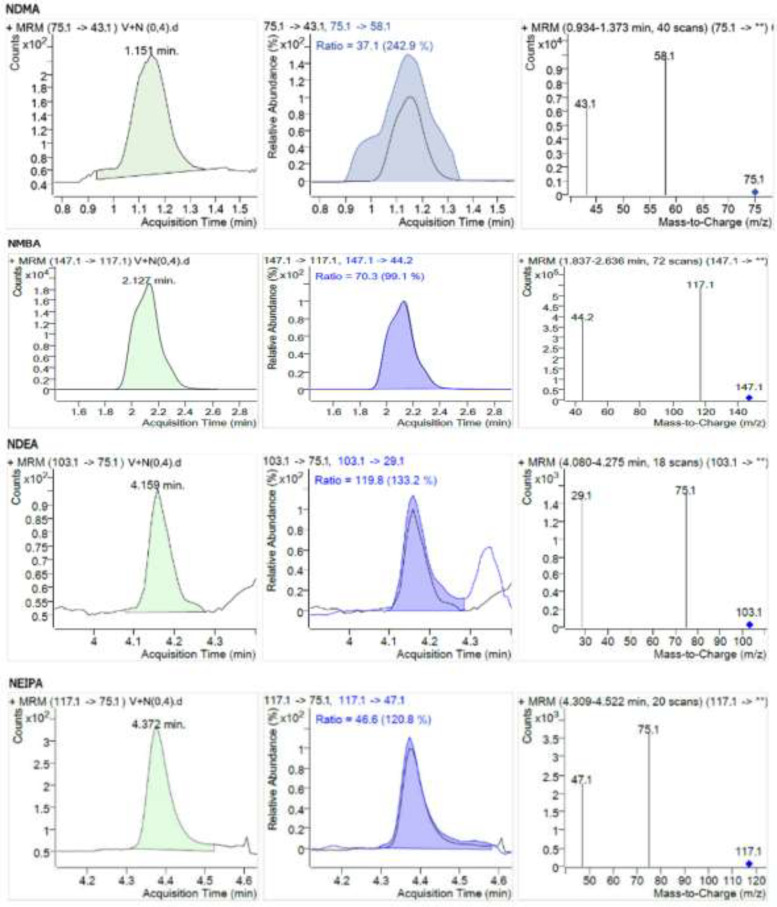
Chromatogram of Valsartan model mixture with addition of nitrosamines (concentration NDMA, NDEA, NMBA, NEIPA: 0.4 ng/mL).

The limit of quantification of the method was obtained with the analysis of model mixtures with the concentrations of 0.2; 0.4; 0.6; 0.8; and 1.1 ng/mL. The acceptance criterion for this parameter was a signal-to-noise (S/N) ratio of 10. Experimentally the limit of quantification was set to 0.4 ng/mL. As NMBA impurity ionizes significantly better than the other impurities under discussion, its limit of quantification is considerably lower than the mentioned value. No studies of method sensitivity for NMBA impurity were conducted. Model chromatograms of nitrosamine impurities with a concentration of 0.4 ng/mL are given in Figure 4.

*Linearity*

Method linearity was assessed with a sequential analysis of model mixtures of pharmaceutical substances (Valsartan, Losartan, and Irbesartan) with nitrosamine concentrations of 0.4; 0.6; 0.8; 0.9; and 1.1 ng/mL. The primary acceptance criterion for the method was correlation coefficient R^2^ > 0.99. Linearity was determined as the function y = ax + b. For linear dependence, mean values obtained with three consequential measurements were used. The equation and correlation coefficient for each nitrosamine in the pharmaceutical substances are given in Table 3.

*Accuracy*

Method accuracy was assessed using recovery of 80 – 120 %. RSD between parallels should be NMT 10%. Recovery is calculated using $R=\frac{\mathrm{Recovered}}{\mathrm{Injected}}\times 100\%$. “Injected” value was calculated using external calibration: nitrosamine impurity standards with the concentrations of 0.4; 0.6; 0.8; 0.9; and 1.1 ng/mL were analyzed. Method recovery is summarized in Table 4.

*Precision*

To determine the precision of the suggested method, repeatability results were assessed. For the test, nitrosamine concentrations in model mixtures were 0.4 ng/mL. The primary acceptance criterion for repeatability was RSD ≤10%.

*Determination of nitrosamine impurities in substances purchased from Chinese manufacturers*

**Figure 5:**
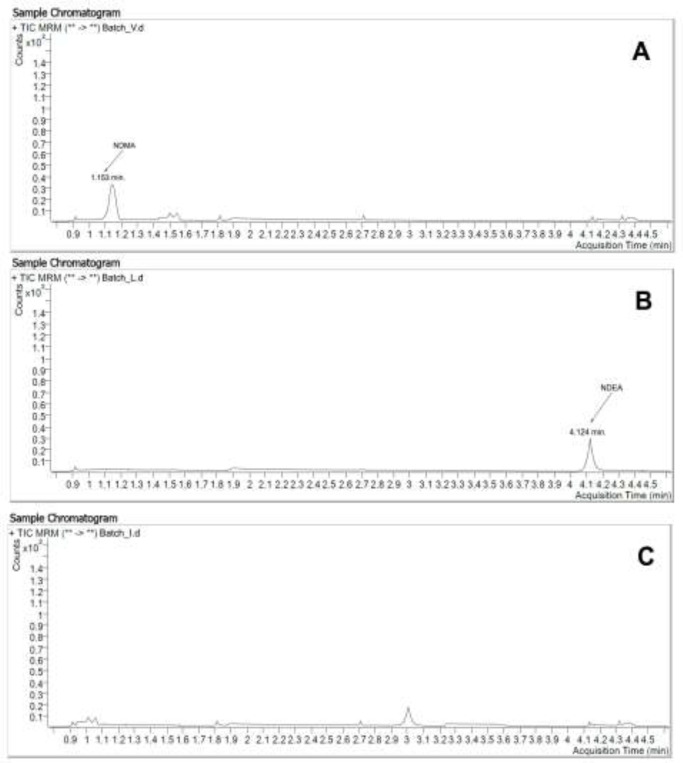
(A) Chromatogram of Valsartan substance. NDMA < 0,4 ng/mL. NDEA, NMBA, NEIPA – not founded. (B) Chromatogram of Losartan substance. NDEA < 0,4 ng/mL. NDMA, NMBA, NEIPA – not founded. (C) Chromatogram of Irbesartan substance. NDMA, NDEA, NMBA, NEIPA – not founded

By the developed method was studied for one batch of both Valsartan, Losartan, and Irbesartan substances. These substances were obtained from Chinese manufacturers, described in Materials and methods. The obtained results are described in Figure 5. 

How it was shown in Figure 5 the developed method allows us to determine low amounts of nitrosamine impurities in the valsartan, Losartan, and irbesartan substances. 

The comparison of the developed method with available FDA and EMA methods shows the following advantages. One of the developed method’s main advantages is the highest sensitivity - 0,2 ng/mL LOQ for all determined nitrosamines (NDMA, NDEA, NMBA, NEIPA). Method FDA has only 3 ng/mL – LOQ for six nitrosamines, including two nitrosamines that are not regulated by the regulatory authorities (16). Moreover, this method includes using rare and expensive equipment (LC-ESI-HRMS) and is less suited to routine quality control than the developed method. Other FDA methods are presented by GC-MS methods. GC-MS (headspace) method has lower LOQ and requires to use of additional equipment (Headspace) (17). The method of GC-MS with direct injection is able to the simultaneous determination of 10 nitrosamine impurities. It has lower LOQ, a much longer time of analysis, and has a risk of column clogging by the high value of the valsartan drug substances (18). Moreover, GC-MS methods are not able to determine nitrosamine in losartan and irbesartan substances. Comparison of the developed method with EMA LC-MS/MS shows the following advantages. Despite the method, EMA has the same sensitivity 0,2 ng/mL, it can determine only 2 nitrosamines (NDMA, NDEA) for one analysis (19). Other EMA methods of nitrosamine determination detect only 1 nitrosamine or have a much lower LOQ (20).

The comparison shows us that the developed method has various advantages. The main advantages are a higher sensitivity than FDA methods available in the literature and less expensive equipment.

**Table 1:** Preparation of calibration standards for impurities

**Solution**	**Volumetric flask capacity** **(mL)**	**Standard aliquot volume (µL)**	**Final concentration (ng/mL)**
Calibration standard 1	100	100	0.2
Calibration standard 2	100	200	0.4
Calibration standard 3	100	300	0.6
Calibration standard 4	100	400	0.8
Calibration standard 5	100	450	0.9
Calibration standard 6	100	550	1.1

**Table 2:** Chromatographic separation parameters and settings of mass spectrometry detection

**Gradient elution parameter**								
**Time (min)**			**Mobile phase А, (%)**		**Mobile phase B, (%)**			
0			95		5			
0.5			95		5			
1			50		50			
1.5			5		95			
5			5		95			
**Other analysis settings**								
Column temperature					50 С			
Flow rate					0.4 mL/min			
Mobile phase A					0.1% formic acid in 10 мМ ammonium formate aqueous solution			
Mobile phase B					0.1% formic acid in methanol			
Injection volume					8 µL			
Stop time					5 min			
Post time					3.5 min			
**MS configuration and source settings**								
Type of ionization source					APCI (atmospheric-pressure chemical ionization)			
Drying gas temperature					350 ºС			
APCI heater					350 ºС			
Drying gas flow rate					6 L/min			
Nebulizer pressure					40 psi			
Capillary voltage, positive					4,000 V			
APCI needle positive					4 µA			
Time frame – NDMA					0.986 – 1.556 min			
Time frame – NMBA					1.845 – 2.711 min			
Time frame – NDEA					4.082 – 4.284 min			
Time frame – NEIPA					4.300 – 4.597 min			
**Retention time and characteristic MRM transitions for nitrosamines under discussion**								
	Retention time (min)	Precursor ion (m/z)		Fragmentor Voltage (V)	Product ion 1 (Qualifier) (m/z)	Collision energy (V)	Product ion 2 (Quantifier) (m/z)	Collision energy (V)
NDMA	1.168	75.1		30	43.1	17	58.1	13
NMBA	2.190	147.1		55	44.2	12	117.1	8
NDEA	4.156	103.1		75	75.1	9	29.1	13
NEIPA	4.380	117.1		50	47.1	17	75.1	17

**Table 3:** Linearity equation and correlation coefficient for nitrosamines

	**Valsartan model mixture**	**Losartan model mixture**	**Irbesartan model mixture**
	Linear dependence equation and correlation coefficient
NDMA	y = 0.0043x + 0.0046R² = 0.9985	y = 0.0043x - 0.0395R² = 0.9989	y = 0.0042x + 0.2838R² = 0.9974
NDEA	y = 0.0043x - 0.0254R² = 0.9989	y = 0.0043x - 0.0222R² = 0.9986	y = 0.0041x + 0.4656R² = 0.994
NMBA	y = 3^.^10^-5^x + 0.1197R² = 0.9989	y = 3^.^10^-5^x - 0.0175R² = 0.9977	y = 3^.^10^-5^x + 0.7113R² = 0.9941
NEIPA	y = 0.0043x - 0.0822R² = 0.998	y = 0.0043x + 0.0194R² = 0.9992	y = 0.0003x + 0.7113R² = 0.9941

**Table 4:** Method recovery

**Pharmaceutical substance**		**Valsartan**		**Losartan**		**Irbesartan**	
Nitrosamine	Injected (ng/mL)	Recovered (ng/mL)	R, (%)	Recovered (ng/mL)	R, (%)	Recovered (ng/mL)	R, (%)
NDMA	0.4	0.392	97.8	0.389	95.8	0.394	98.3
		0.389		0.376		0.391	
		0.393		0.385		0.372	
	0.6	0.591	97.2	0.581	96.1	0.594	95.4
		0.586		0.584		0.541	
		0.574		0.566		0.557	
	0.8	0.811	97.5	0.819	95.6	0.820	95.6
		0.744		0.731		0.768	
		0.787		0.745		0.739	
NDEA	0.4	0.375	97.5	0.390	95.5	0.395	97.3
		0.368		0.384		0.347	
		0.427		0.372		0.353	
	0.6	0.568	90.3	0.642	93.7	0.538	95.9
		0.542		0.510		0.574	
		0.516		0.536		0.523	
	0.8	0.764	93.1	0.777	97.5	0.715	101.1
		0.749		0.764		0.821	
		0.723		0.801		0.782	
BMSA	0.4	0.396	98.3	0.391	98.5	0.389	98.5
		0.393		0.395		0.388	
		0.391		0.396		0.394	
	0.6	0.596	99.1	0.594	99.1	0.591	99.1
		0.597		0.595		0.589	
		0.592		0.595		0.593	
	0.8	0.790	99.2	0.794	99.3	0.794	100.7
		0.799		0.793		0.795	
		0.792		0.798		0.799	
NEIPA	0.4	0.376	93.5	0.383	91.9	0.395	95.7
		0.364		0.376		0.348	
		0.383		0.344		0.337	
	0.6	0.582	94.3	0.594	94.8	0.546	94.3
		0.569		0.548		0.559	
		0.547		0.566		0.543	
	0.8	0.786	97.2	0.737	90.7	0.720	94.9
		0.790		0.722		0.771	

**Table 5:** The precision of the HPLC-MS/MS method

** Replication**		**1**	**2**	**3**	**4**	**5**	**6**	**RSD (%)**
**Description**								
NDMA	Peak area	829	918	910	924	872	907	4.06
NDEA		931	814	930	800	875	929	6.87
NMBA		120070	119399	123274	125207	124018	123027	1.86
NEIPA		830	927	902	925	810	957	6.57

## Conclusions

The method developed for determining nitrosamines in Valsartan, Losartan, and Irbesartan pharmaceutical substances was validated for all significant parameters. It was found out that the method possesses the required sensitivity, selectivity, linearity, and accuracy. Selectivity was determined with the lack of foreign peaks in the area of nitrosamine detection. Linearity is 0.4 to 1.1 ng/mL for NDMA, NDEA, NMBA, NEIPA. A correlation coefficient is over 0.99. Method accuracy was assessed using recovery; all values are within 80-120%. Method precision complies with the acceptance criterion of RSD < 10%.

Chemical ionization at atmosphere pressure allowed achieving the highest sensitivity, while correct and selective MRM transitions have eliminated matrix effects. As a result, no isolation and concentration are required during sample preparation. Method sensitivity for nitrosamines expressed in terms of the substance was: 

0.0125 ppm for Valsartan

0.0260 ppm for Losartan

0.0130 ppm for Irbesartan

The sensitivity is almost seven times lower than the currently recommended level. The method can be used for routine quality control of pharmaceutical substances (Valsartan, Losartan, and Irbesartan) for nitrosamine impurities.

## References

[1] White CM (2020). Understanding and Preventing (N-Nitrosodimethylamine) NDMA Contamination of Medications. Ann. Pharmacother..

[2] Zmysłowski A, Książek I, Szterk A (2020). N-Nitrosodimethylamine Contamination in the Metformin Finished Products. Molecules.

[3] Shen R, Andrews SA (2013). Formation of NDMA from ranitidine and sumatriptan: the role of pH. Water Res..

[4] Liu YD, Selbes M, Zeng C, Zhong R, Karanfil T (2014). Formation mechanism of NDMA from ranitidine, trimethylamine, and other tertiary amines during chloramination: a computational study. Environ. Sci. Technol..

[5] Shaik KM, Sarmah B, Wadekar GS, Kumar P (2020). Regulatory Updates and Analytical Methodologies for Nitrosamine Impurities Detection in Sartans, Ranitidine, Nizatidine, and Metformin along with Sample Preparation Techniques. Crit. Rev. Anal. Chem..

[6] Fjellsbø LM, Verstraelen S, Kazimirova A, Van Rompay AR, Magdolenova Z, Dusinska M (2014). Genotoxic and mutagenic potential of nitramines. Environ. Res..

[7] Charoo NA, Ali AA, Buha SK, Rahman Z (2019). lesson learnt from recall of valsartan and other angiotensin II receptor blocker drugs containing NDMA and NDEA impurities. AAPS Pharm. Sci.Tech..

[8] Shaikh T, Gosar A, Sayyed H (2020). Nitrosamine impurities in drug substances and drug products. J. Pharm. Pract..

[9] Souliotis VL, Henneman JR, Reed CD, Chhabra SK, Diwan BA, Anderson LM, Kyrtopoulos SA (2002). DNA adducts and liver DNA replication in rats during chronic exposure to N-nitrosodimethylamine (NDMA) and their relationships to the dose-dependence of NDMA hepatocarcinogenesis. Mutat. Res..

[10] Taniguchi M, Yasutake A, Takedomi K, Inoue K (1999). Effects of N-nitrosodimethylamine (NDMA) on the oxidative status of rat liver. Arch. Toxicol..

[11] Chao MR, Hsu YW, Liu HH, Lin JH, Hu CW (2015). Simultaneous Detection of 3-Nitrotyrosine and 3-Nitro-4-hydroxyphenylacetic Acid in Human Urine by Online SPE LC-MS/MS and Their Association with Oxidative and Methylated DNA Lesions. Chem. Res. Toxicol..

[12] Plumlee MH, López-Mesas M, Heidlberger A, Ishida KP, Reinhard M (2008). N-nitrosodimethylamine (NDMA) removal by reverse osmosis and UV treatment and analysis via LC-MS/MS. Water Res..

[13] Topuz E, Aydin E, Pehlivanoglu-Mantas E (2012). A Practical LC-MS/MS Method for the Detection of NDMA at Nanogram per Liter Concentrations in Multiple Water Matrices. Water Air Soil Pollut..

[14] Masada S, Tsuji G, Arai R, Uchiyama N, Demizu Y, Tsutsumi T, Abe Y, Akiyama H, Hakamatsuka T, Izutsu KI, Goda Y, Okuda H (2019). Rapid and efficient high-performance liquid chromatography analysis of N-nitrosodimethylamine impurity in valsartan drug substance and its products. Sci. Rep..

[15] Parr MK, Joseph JF (2019). NDMA impurity in valsartan and other pharmaceutical products: Analytical methods for the determination of N-nitrosamines. J. Pharm. Biomed. Anal..

[16] Liquid Chromatography-High Resolution Mass Spectrometry (LC-HRMS) Method for the Determination of Six Nitrosamine Impurities in ARB Drugs.

[17] GC/MS Headspace Method for Detection of NDMA in Valsartan Drug Substance and Drug Products.

[18] Combined Direct Injection N-Nitrosodimethylamine (NDMA), N-Nitrosodiethylamine (NDEA), N-Nitrosoethylisopropylamine (NEIPA), N-Nitrosodiisopropylamine (NDIPA), and N-Nitrosodibutylamine (NDBA) Impurity Assay by GC-MS/MS.

[19] Method for the determination of NDMA and NDEA by LC-MS/MS in Sartans (drug substance and film coated tablets).

[20] Determination of NDMA and NDEA in SARTAN drug substances by HPLC/UV.

